# The Development of Understanding Opacity in Preschoolers: A Transition From a Coarse- to Fine-Grained Understanding of Beliefs

**DOI:** 10.3389/fpsyg.2020.00596

**Published:** 2020-04-07

**Authors:** Arkadiusz Gut, Maciej Haman, Oleg Gorbaniuk, Monika Chylińskia

**Affiliations:** ^1^Department of Cognitive Science, Nicolaus Copernicus University in Toruń, Toruń, Poland; ^2^Department of Philosophy, The John Paul II Catholic University of Lublin, Lublin, Poland; ^3^Faculty of Psychology, University of Warsaw, Warsaw, Poland; ^4^Institute of Psychology, University of Zielona Góra, Zielona Gora, Poland; ^5^Institute of Psychology, The John Paul II Catholic University of Lublin, Lublin, Poland

**Keywords:** opacity, theory of Mind cognitive development, intensionality, false belief task, childhood, cognitive development

## Abstract

Intensionality (or opacity) is a core property of mental representations and sometimes understanding opacity is claimed to be a part of children’s theory of mind (evidenced with the false belief task). Children, however, pass the false belief task and the intensionality tasks at different ages (typically 4 vs. 5;1–6;11 years). According to two dominant interpretations, the two tests either require different conceptual resources or vary only in their executive or linguistic load. In two experiments, involving 120 children aged 3–6 (Experiment 1) and 75 children aged 4–6 (Experiment 2), we tested two variants of the executive load hypothesis: The differential linguistic complexity of the two tests, and the dual-name problem of the intensionality task. The former was addressed by standardizing and minimizing the linguistic demands of both tasks (contrasted with the typical narrative intensionality task), and the latter by introducing the dual-name problem into the false belief task as well, so that it was present in both tasks. We found that (1) two structurally different intensionality tasks shared more variance with each other than with the structurally similar false belief task, and that (2) introducing a dual label problem into the false belief task did not reduce the developmental gap. Our results speak against interpreting the difference between the time children pass the two tests entirely in terms of performative issues, and support the conceptual enrichment hypothesis. We discuss the theoretical relevance of these results, suggesting that they are best explained by fine-grained increments within the concept of belief, rather than a radical conceptual change. We conclude that understanding opacity of minds – which emerges between age 5 and 6 – is an important step toward a more advanced form of ToM.

## Introduction

When we hold a belief, we always necessarily do so under a specific term. This fact has been recognized in philosophy and cognitive psychology, and has been studied under the label of *opacity* or *intensionality* of mental representation (Leslie, 1987; Baron-Cohen, 1996; Mitchell, 1996; Doherty and Perner, 1998; Gopnik et al., 1999; Kamawar and Olson, 1999, 2011; Apperly and Robinson, 2001, 2003; Hulme et al., 2003; Perner et al., 2003, 2015; Sprung et al., 2007; Fabricius et al., 2010; Apperly, 2011; Wellman, 2014; Rakoczy et al., 2015; Rakoczy, 2017)^1^. In this paper we investigate whether understanding opacity develops together with understanding false beliefs (evidenced by passing the *false belief test –* FBT) as a part of the standard *theory of mind* (ToM), or whether the two require distinct conceptual constructs.

Although at first it was assumed that false-belief understanding and understanding intensionality or opacity were provided by the same ToM mechanism (see, for example, Perner, 1989, 2000; Flavell, 1993; Gopnik, 1993; Mitchell, 1996; Doherty and Perner, 1998; de Villiers and Pyers, 2002; Perner et al., 2002, 2003; Perner and Roessler, 2012), later research revealed a clear age difference in passing FBTs and intensionality (opacity) tasks (IT): Performance on IT at six is comparable to performance on FBT at four (see, for example, Russell, 1987; Kamawar and Olson, 1999, 2009, 2011; Apperly and Robinson, 2001, 2003; Hulme et al., 2003). However, the reason for the difference is still disputed.

There are two main interpretations of the observed age gap between FBT and IT: (i) First is that genuine conceptual deficits lie behind the children’s performance (ii) Second is that only some executive and linguistic factors make IT more difficult than FBT, with conceptual understanding needed for both always being there, but unexpressed (Rakoczy, 2012; Rakoczy et al., 2015). Below we revise previous studies investigating these hypotheses, offer some critique, and propose our own position, which we investigate empirically with the present study.

Let us first discuss the possible *conceptual* differences between FBT and IT. Consider this fundamental point about intensionality first. From a psychological perspective, extensional (transparent) contexts (“Cicero was Roman”) need to be distinguished from intensional (opaque) ones (“S believes that Cicero was Roman”) (Quine, 1960/2013). Ascribing a belief to someone, we try to envisage the individual’s way of representing the object. Even though Cicero and the author of *Pro Quinctio* are the same person, one may not know that, and effectively believe that Cicero was Roman without believing that the author of *Pro Quinctio* was Roman (Apperly, 2011; Quine, 1960/2013). In contexts such as these, we cannot respect the principle of extensionality: We cannot substitute the term “the author of Pro Quinctio” for the term “Cicero” and rely on the standard relations of truth between the statements (Astington and Jenkins, 1999; Baron-Cohen, 1996; Quine, 1960/2013).

Understanding intensionality of representation is therefore clearly more complex conceptually than understanding only its referential truth value. FBT measures the latter, but not the former. To understand that people can hold false beliefs requires one to consider that they can represent the wrong referent. That is, false belief attribution can be entirely within the realm of reference and its correctness or incorrectness, and need not involve any consideration of *how* a given person represents the referent. Understanding intensionality or opacity, on the other hand, requires one to consider the mode of representation (intension). If one is to understand intensionality, one needs to appreciate the fact that people always represent referents under particular concepts (intensions), and that the intension under which something is believed makes a systematic difference for how people act and what other beliefs they hold.

Consequently, the conceptual-deficit interpretation of the age gap is that children at first cannot differentiate intension from reference; that is, they cannot understand that beliefs not only differ in relation to what they are about (that is a situation in the world), but also to the mode of representing the situation in the world. We discuss proposals that espouse this interpretation below.

Apperly and Robinson (2003) rightly argue that the higher difficulty of ITs relatively to FBTs (in ages 4–7) cannot be simply explained by the child’s inability to understand that beliefs may vary among individuals with the very same informational access, or by their lack of skill in handling ambiguous utterances. Apperly and Robinson (2003) do not, however, specify what exactly needs to change in the child’s conceptual understanding for the child to pass IT as reliably as they pass FBT. They limit their claim to a suggestion “that the representational nature of mental states should be viewed as posing more than one distinct problem, which children may solve in distinct ways and at different points in development” (2003, p. 308; cf. Apperly, 2011). Another important – and in our opinion, right – claim that Apperly and Robinson make is that understanding IT is not about understanding different informational access (e.g., one identity of an object is acquired through physical manipulation, and another through a narrative about it). In our experiment, we demonstrate that how an identity of an object was learned does not impact the child’s performance on IT.

Further, our proposal is largely consistent with that of Kamawar and Olson (1999), but with important differences. We agree with the claim that “passing false belief tasks and being able to deal appropriately with opaque contexts are manifestations of a developing understanding of representation more generally” (1999, 545). Their main position is that a two-part (representation-referent) model of representation suffices for passing FBT, but IT requires the child to adopt a three-part model (representation-sense (intension)-reference). However, we are much less certain than Kamawar and Olson that such a paradigm-shift transition from a two- to three-part model of representation is the right interpretation. We believe that even when the child passes FBT only, she to some extent understands representations in a three-part way. The central problem by our lights is that prior to passing the FBT, the child’s understanding of intensionality is closely related to their understanding of reference – they understand intension in terms of logical value or the correctness of reference. This understanding of intension/sense works for FBT, but is not sufficient for IT, where she needs to appreciate that intensionality is importantly different from reference.

We argue that the gap in performance involves a conceptual enrichment that consist in expanding the content of particular concepts (e.g., “belief”), enabling the child to add new, more sophisticated principles of mental states attribution to already existing abilities. We term it a transition from coarse-grained content to fine-grained content. Such a hypothesis leads to the following interpretation: False-belief reasoning and opacity reasoning both involve taking multiple perspectives – i.e., the category of *sense* – but in FBT, “sense” consists of epistemic values (true-correct vs. false-incorrect), while in IT, of epistemic perspective (intensionality or opacity). Children have to realize the new dimensions of beliefs understanding and start to comprehend that having access to an object under one description does not ensure access to that object under all descriptions. Further enrichments in the conceptual toolbox of the child undoubtedly do not stop at this, but continue and lead to the emergence of a more nuanced, advanced ToM (Carpendale and Chandler, 1996).

Now we turn to the competence-performance interpretations of the age gap. One performance-based problem that could explain the age gap between passing FBT and IT may be difficulty in substituting co-referentials in intensional contexts, which may stem solely from the test’s load on executive functions, or linguistic complexity. The problem with multiple terms for a single referent in the process of first-language acquisition has been known since Markman’s work on “mutual exclusivity hypothesis” (Markman, 1989), which states that the implicit assumption of one-to-one name-to-referent mapping is a constitutive part of children’s mechanisms of lexical development.

Another possibility points to the linguistic complexity of the stories and the tests used in the experiments described above. The test questions involved multiple embedded clauses, which also may impose too heavy a load on the child’s capacities of processing linguistic input (Robinson and Mitchell, 1992; de Villiers and Pyers, 2002; Perner et al., 2002; Sprung et al., 2007; Kamawar and Olson, 2011; Rakoczy et al., 2015; Rakoczy, 2017).

Although these problems have been partially addressed by two previous studies (Hulme et al., 2003; Rakoczy et al., 2015), we believe they leave some issues unresolved. Hulme et al. (2003) designed a study to test the hypothesis that the nature of the problems with intensionality is linguistic rather than conceptual. They used pictures that the child had to choose from, instead of giving verbal answers. The participant was shown a set of pictures which displayed: an outline of a body with a question mark on the torso; a prototypical image of a teacher (with glasses and with hair tied in a bun); a picture of the actual teacher (as presented in the story); and a distracter picture of a person who looked unlike anyone relevant to the situation. All of the presented figures had a whistle. The participant was asked to select the picture which best fit what the protagonist – Rosie – was thinking. Rosie was introduced as a girl who has just started school and does not know anybody yet. In the condition where only the participant had been shown the picture of the actual teacher, most of the children answered correctly the knowledge question (i.e., *“Does Rosie know what the person who has the whistle looks like?”*), and thus concluded that Rosie did not know what the person who had the whistle looks like. It could be expected then that children would at least avoid selecting the picture of the real teacher in the intensionality question (i.e*., “Choose the picture that best fits what Rosie is thinking.”*), or may select either the prototypical or the outline picture. Nine-year-old children chose almost exclusively the conventional body shape with a question mark. Conversely, 6-year-olds selected the picture of the actual teacher. Importantly, in another condition when they did not know the identity of the teacher, they choose most frequently the picture of a prototypical teacher.

Unfortunately, although Hulme et al. (2003) replicated this result in a series of three follow-up studies, designed to test some alternative explanations, the task is far from conclusive. First, the study addressed only the problem of the mode of the response: The verbal answer was replaced by choosing one of several pictures. The rest of the task was presented verbally, and the stories used in the study were relatively complex. Second, understanding that people are guided by stereotypes when lacking detailed knowledge and that we can represent lack of knowledge by a conventional sign (a body outline with a question mark) goes well beyond a simple theory of mind and has little to do with opacity itself. Third, the test question could have been ambivalent from the child’s perspective: Even though Rosie did not know exactly what the teacher looked like, the child could assume that Rosie would correctly identify the person as a teacher on the basis of information that *was* available to her – that the person was a P.E. teacher, had a whistle, and would come out of the opposite room. And finally, Hulme et al. (2003) did not address the problem of co-referentials at all, but only how intension relates to reference: The tasks do not involve identifying a person as one of two possible identities – e.g., as a sister or doctor – but only correctly identifying them as actually being or not being the referent of the one identity in question (the teacher).

We are also critical of Hulme et al.’s claim that difficulties on IT stem from the inability to distinguish whether the content of the representation (belief) involves an *individual concept* or a *descriptive concept*. As mentioned above, we believe after Apperly and Robinson that informational access is not a good candidate for how the child understands an object’s different identities and their representation by other people. We did, however, control for that in our study: children acquired the knowledge of different identities in two different ways (physical manipulation and narrative).

More recently, Rakoczy et al. (2015) introduced an innovative version of the intensionality test, based on the unexpected-change-of-location FBT. In the first experiment 4- to 5-year-old children were familiarized with an object X having a double identity (e.g., a soft toy that could be turned inside out and was a bunny on one side and a carrot on the other side) or double property (e.g., a blue sock becoming red after reversing). The object X was hidden in a box A in the presence of the protagonist, then turned into the second identity/property in the absence of the protagonist, and transferred to the box B after the protagonist returned. The child was then asked were the protagonist will look for X under the former aspect (e.g., bunny or blue sock). Typical control and memory questions were also asked, and two groups of control questions were added: extensional questions and true belief questions. All groups also solved FBT based on Wimmer and Perner’s (1983) procedure. Rakoczy et al. have reported that most of the children who passed FBT, passed the intensionality test as well.

Although the idea of the test itself seems appropriate, we are not convinced that the results provide enough support to the thesis that early belief understanding involves understanding opacity. Firstly, the sample consisted of 4- to 5-year-olds, i.e., a mixture of children who, according to other studies, are at the age when understanding beliefs (age of four) and understanding intensionality (age of five) start. Since the experimental groups were small (*N* = 20), authors reported the results for whole groups only. Additionally, the proportion of the children passing FBT was higher in the intensionality condition than in both control groups (75 vs. 55 and 50 percent, respectively). Moreover, one of the details of the procedure also raises our concerns. In the intensionality test scenario, children were told that turning the object identity/property is “playing a trick” on the protagonist. Although used also in some other versions of FBT, this procedure is questionable. Before acquiring a fully-fledged ToM, young children may possess functional understanding of “a trick” (deception), assuming that deceived protagonist will behave contradictory to the reality (cf. Ruffman et al., 1993). Thus, if the child knows that X is in the box B, he/she may expect the protagonist to search for X in the box A by simply assuming that the adult experimenter who helped with the trick was good at playing tricks on others^2^.

The present study attempts to address the issues of the above experiments and provide a more definite answer to the question whether the gap between false belief understanding and intensionality understanding is conceptual or only performational in nature. We designed tasks that reduced both executive and linguistic loads of intensionality tests and warranted their full compatibility with FBT. We did this so that if the time gap persisted, it would strongly suggest a genuine conceptual difference between the two kinds of understanding.

## Current Study

We designed two multitask experiments. Experiment 1 aimed at replication and validation of the results reporting a lag between FBT and IT. However, we introduced some important changes that distinguish our study from the previous ones. Firstly, we decided to use the unexpected contents task (Hogrefe et al., 1986; Perner et al., 1987) instead of the change-of-location version used in most of the previous studies (e.g., “Sally and Ann”). The logic of the unexpected contents task is analogous to the typical intensionality test as both of them appeal to the identification of the object as a particular thing (e.g., dad or police officer), not the object’s location. Thus, it is easy to construct equivalent tasks for both tests. Secondly, we designed two versions of IT: (1) the standard narrative, in which all information was provided verbally as a story, and (2) the task in which the crucial information was available through visual inspection and manipulation of the object. Thirdly, according to the simulation theory (Goldman, 2008), estimation of one’s own, first-person mental states may be easier to manage than third-person ones. If so, asking a first-person question may reduce complexity of the task and thus reduce the time lag between FBT and IT.

Experiment 2 was designed mainly to falsify the hypothesis that double names referring to the same object in IT explains why children pass it later than FBT. In other words, we wanted to show that even when controlling for the linguistic load, children pass the two tasks at different times in development. To do that, we eliminated the need for verbal reference to the object identity. Similarly to Hulme et al.’s (2003) task, we applied pictorial representations. The child’s task was now to choose a subset of stickers (from a set of two or four) which could stand for the object identity known to the protagonist in both FBT and IT. In the four-sticker set, two different stickers represented each of the object’s identities, so if it was the double names that caused the relative difficulty of IT, the four-sticker condition would additionally impair both FBT and IT performance. On the other hand, if the problem was generally at the level of linguistic requirements of the response rather than the conceptual level, using stickers instead of the object’s names should reduce the delay between passing FBT and IT. Finally, if understanding opacity was conceptually more complex than false belief understanding (as stated in our hypotheses), the results of the sticker version of the experiment should parallel standard, verbal versions (i.e., the ones used in Experiment 1).

We expected that our study would allow us to explore how understanding intensionality develops in pre-school children. Further, we hoped to solve the problem of whether understanding that mental representations may be false involves also understanding that mental representations are opaque. Additionally, we would touch upon the issue of the difficulty of coping with understanding intensionality of mental representations connected with the use of two labels for the same object. We predicted that using two labels for the same object was not the reason why IT was more difficult than FBT, but that an actual conceptual difference explained the disparity: The standard theory of mind acquired at the age of four has to be enriched by understanding opacity if the child is to pass IT.

## Experiment 1

### Method

#### Participants

One hundred and twenty children, aged from 40 months to 83 months, participated in the study. The participants formed four age groups of 30 children: 3-year-olds (mean age 3;8, range 3;4–3;11), 4-year-olds (mean age 4;7, range 4;2–4;11), 5-year-olds (mean age 5;6, range 5;1–5;11), and 6-years-olds (mean age 6;6, range 6;1–6;11). Participants came from middle-income urban families from three different kindergartens in an average-size city in Poland.

#### Tasks and Procedure

Three tasks were administered to all participants: the unexpected content task version of FBT (based on the “Smarties task”), and two intensionality tasks designed for the purposes of the current study.

##### False belief task

At the beginning of the task, two experimenters introduced themselves to the child. Then, the experimenters and the child sat at the table in the room. A popular box of eight Kinder chocolate bars was used. The pack was new and looked as if it had not been opened. Instead of chocolates, there were colored pencils inside it. The first experimenter checked whether the child was familiar with the chocolates, asking who bought the chocolates in the child’s house and whether the child liked them. A control question was also asked: *“Do you know what’s usually in the box?”*. After an answer, the first experimenter and the child participant remained in the room whereas the second experimenter left the room, saying that she would be back in a while. After the second experimenter had left the room, the first experimenter showed the content of the box to the participant, and it turned out that there were pencils in the box, not chocolates. Then, the experimenter asked the child the test question: *“Does X who has left the room know that there are pencils in the box?”* (where X stands for the name of the second experimenter). We modified the standard FBT question and used the form of “Does X who has left the room know/think that there are pencils in the box?”. The motivation for this was to reduce performance differences of FBT and IT by making the questions in both syntactically identical. In the pilot studies, we used “think” and “know” interchangeably in the test questions. There were no essential differences between the two conditions, but because of the specificity of the language in which the study was conducted, we used the verb “know” in FBT questions^3^. A first-person test question was also asked*: “Did you know before I showed you what was inside the box, that there were pencils in the box?”* If the child understood false beliefs the answer to both questions should have been *“no”.*

##### Intensionality tasks

There were two ITs. In one of the tasks, children received the needed knowledge through play (manipulation and visual inspection); they alternated between playing with the object first as a car and then as a pen. The other task conveyed the information in the form of a narrative about a person who is a police officer and a dad at the same time.

##### Car-pen task

Two experimenters introduced themselves to the child, and then they sat at the table in the room. A plastic toy car that was also a ballpoint pen was used; the pen was hidden and would slide out after pressing a button (see Figure 3). The first experimenter took out the car from a black box. The child could touch the car and play with it for a minute. The second experimenter participated in the play. Next, the first experimenter put the car back into the box, and the second experimenter left the room, saying that she would be right back. After that, the experimenter took out the car again, telling the child that she was going to show him/her something extra. The experimenter pushed a hidden button on the car, which made the pen slide out. The child could draw something with it on a sheet of paper, after which the experimenter made the pen slide back in and put the car-pen back into the box. Then, the first experimenter asked the child two questions: “*Does X, who has left the room, know that there is a pen in the box?”*, and *“Does X, who has left the room know that there is a car in the box?”* (where X stands for the name of the second experimenter). The first question was the main test of opacity understanding. If the participant understood opacity, he/she would answer *“no.”* The second question was the main control question. If the child had correctly assigned knowledge to the protagonist, he/she would answer *“yes.”* These two questions were asked in random order. Then two extensional questions were asked (also in random order) to test if the child correctly represented the double identity of the object: *“Is there a car in the box?”*, and *“Is there a pen in the box?.”* For both of these questions the correct answer was *“yes”.* Finally, always as the last one, a first-person question testing opacity understanding was asked.

##### Police officer-dad task

The same initial arrangements as in the previous tasks were used. The first experimenter told the child a story:

Imagine there is a girl called Anna who always has to cross a very busy street when she gets back from school. One day, when Anna was getting back from school, it turned out that the traffic signals were not working. A police officer was directing the traffic in the street. Anna had been standing at the crossing because there were a lot of cars and she was afraid to cross to the other side. The police officer noticed Anna and walked up to her. He took her by the hand and led her to the other side of the street.

That was where the experimenter stopped the story. The second experimenter, who had been also listening to the story until this moment, now left the room, saying that she would be right back. Then the first experimenter told the child that she would tell him/her something extra about the story. This extra information was that the police officer was in fact Anna’s dad. After that, a series of questions analogous to the car-pen task, and ordered according to the same rules, were asked: Intensionality test question: *“Does X, who has left the room, know that Anna was taken to the other side of the road by her dad?”.* Control question: *“Does X who has left the room know that Anna was taken to the other side of the road by the police officer?.”* Extensional questions: *“Was Anna taken to the other side of the road by a police officer?”* and *“Was Anna taken to the other side of the road by her dad?.”* First-person intensionality test question: *“Did you know that Anna was taken to the other side of the street by her dad before I told you so?”*

The same scoring rules as in the car-pen task were applied in the police officer-dad task.

Children were tested in a room in their schools. In approximately half of the cases, FBT was administered first, followed by two ITs in random order. In the remaining cases, IT was administered as the first task.

#### Statistical Analyses

In the current study and the studies that follow, we first examined differences between age groups in answers separately for each task (one score for correct answer and zero for wrong answer), using χ^2^ test and Cramer’s V as a measure of effect size for these differences. Next, the differences in the proportions of correct answers between the two questions in the same group were tested by McNemar χ^2^ and between-task correlation by Yule’s φ. The differences in the proportions of the correct answers between more than two questions were tested by Cochran’s Q. Finally, we conducted a hierarchical logistic regression to control the influence of age on the task performance as dependent variables, and to test how adding additional predictor variables allows for explaining task performance. The statistical software IBM SPSS Statistics (version 25.0) was used for analyses. The significance level for the studies was 0.05.

### Results

#### False Belief Task

There was a clear age-related increase in the number of children passing FBT (see Table 1): from eight children (28%) in the 3-year-old group, to 27 children (90%) in both 5- and 6-year-olds [χ^2^(3) = 36.885; *p* < 0.001, Cramer’s *V* = 0.554]. These results are consistent with the previous reports (Wellman et al., 2001).

**TABLE 1:** Distribution of correct answers to false belief and intensionality tests, and control questions.

	Age				Total
Test	3-year-olds	4-year-olds	5-year-olds	6-year-olds	
IT1 (test question) Third-person	3(10.0%)	11(36.7%)	19(63.3%)	26(86.7%)	59(49.2%)
IT2 (test question) Third-person	4(13.3%)	12(40.0%)_f_	20(66.7%)	28(93.3%)	64(53.3%)
FBT(test question) Third-person	8(26.7%)	19(63.3%)	27(90.0%)	27(90.0%)	81(67.5%)
IT1 (control question)	27(90.0%)	25(83.3%)	25(83.3%)	25(83.3%)	102(85.0%)
IT2 (control question)	26(86.7%)	24(80.0%)	27(90.0%)	24(80.0%)	101(84.2%)
IT2 (test question) First-person	2(6.7%)	7(23.3%)	18(60.0%)	24(80.0%)	51(42.5%)
IT1 (test question) First-person	1(3.3%)	5(16.0%)	17(56.7%)	21(70.0%)	44(36.7%)
FBT (test question) First-person	2(6.7%)	14(46.7%)	22(73.3%)	23(76.7%)	61(50.8%)

*FBT, false belief task; IT1, car-pen task; IT2, police officer task.*

#### Intensionality Tasks

Table 1 contains detailed distribution of answers to both intensionality and control questions. A clear age-related near-linear increase in the number of correct answers to the test question was found in both ITs. For both tasks the age-related differences were significant, and the association between task performance and age group was strong [χ^2^(3) = 39.578, *p* < 0.001, Cramer’s *V* = 0.574 in the car-pen task; and χ^2^(3) = 42.857; *p* < 0.001, Cramer’s *V* = 0.598 in the police officer-dad task]. Remember that both tasks differ in their procedures and the way of acquiring the necessary information by the child: in one of them, the information is acquired through play (manipulation and visual inspection) and in the other – it is acquired from a narrative about a person who is a police officer and a dad at the same time. The differences in the proportions of correct answers between the two ITs are small and not significant [McNemar χ^2^(1) = 0.935, *p* = 0.332], and a strong between-task correlation was found (Yule’s φ = 0.719, *p* < 0.001). It seems that a vast majority of children responded in an “all-or-none” mode: Only 17 out of 120 (14.2%) children correctly answered one task but failed another one, which makes it highly probable that both tasks, in spite of salient differences in the procedure, appealed to the same cognitive capacities.

All age groups performed well in the control questions in both tasks (range: 80–90%, see Table 1 for details). There were no significant differences across the age groups [χ^2^(3) = 0.784, *p* = 0.853, *V* = 0.09 for the police officer-dad task; and χ^2^(3) = 1.688, *p* = 0.640, *V* = 0.12 for the car-pen task; all tests were two-tailed]. No correlation between the test and the control question was found (Yule’s φ = 0.04).

Children in all age groups performed near ceiling also in all the extensional questions in both tasks (range: 80–100%). None of the differences between the age groups was significant [χ^2^(3) = 2.069, *p* = 0.558, *V* = 0.14 for the police officer-dad task; and χ^2^(3) = 2.667, *p* = 0.444, *V* = 0.15 for the car-pencil task; all tests were two-tailed]. Again, no correlation between the main test performance (intensional questions) and the extensional question was found (Yule’s φs from 0.01 to 0.13, ns.). The higher level of performance in the control and extensional questions confirms the reliability of both ITs, and proves that the problem of younger children with understanding opacity was not related to retaining and managing the relatively complex information required to solve the problem.

#### Comparison Between Intensional and False Belief Tests

There is a near one-year lag between the ability to pass FBT and IT. Cochran’s *Q* test on correct vs. incorrect answers (FBT vs. ITs) shows a significant difference, with higher proportion of correct answers in FBT than in both ITs [Q(2) = 21.568, *p* < 0.001]. The difference remains significant for both ITs compared separately to FBT [the car-pen task: McNemar χ^2^(1) = 12.971, *p* < 0.001; the police officer-dad task: McNemar χ^2^(1) = 11.130, *p* < 0.001]. Only six out of 120 children (5%) failed FBT but passed IT, and three of them belonged to the oldest group (6-year-olds).

When comparing FBT and IT performance for each age group separately, we found significant differences for all groups except 6-year-olds [Cochran’s Qs(2) = 6.000, 7.500, 10.360, and 0.857, for 3-, 4-, 5-, and 6-year-olds, respectively, all ps < 0.05 except for 6-year-olds, who performed well in all three tasks: *p* = 0.651, ns.].

#### First-Person and Third-Person Questions

The first-person form of the test question did not improve the performance in either of the tests (see Table 1). Rather, some small, but significant decrease in correct answers was observed in first-person questions both in ITs and in FBTs in relation to the third-person question (the car-pen task: 36.7% vs. 49.2%, McNemar χ^2^(1) = 7.840, *p* < 0.01; the police officer-dad task: 42.5% vs. 53.3%, McNemar χ^2^(1) = 6.261, *p* < 0.05; and FBT: 50.8% vs. 67.5%, McNemar χ^2^(1) = 11.281, *p* < 0.001). These results seem to testify against a false-belief and opacity understanding based on simulation. Indeed, despite the small difference mentioned above, the internal consistency of all four questions testing intensionality understanding (two third-person and two first-person) was high (Cronbach’s alpha = 0.88), which affirms that they most probably measure the same general capability.

#### Hierarchical Regression Model

We used hierarchical logistic regression to control the influence of age on the car-pen (model 1) and the police officer-dad (model 2) task performance as dependent variables, and test how adding additional independent/predictor variables allows to explain task performance. These additional variables were the result of the second IT (the police officer-dad task and the car-pen task, respectively) and the first-person question. Our reason for introducing the variables into the model is the following: The control and extensional questions measured whether the child understood the information contained in the task; age explained the variance related to general developmental differences; and FBT result explained the coarse-grained understanding of the representational status of beliefs. At the other end, the first-person question accounted for task-specific variables (e.g., mode of presentation, specific elements of the scenario etc.). The crucial point was including into the model the results of the second IT. If two different ITs appeal to the same conceptual structures, which go beyond the conceptual knowledge required to solve FBT, we may expect that adding the second IT’s results to the model would still explain some substantial part of the variance, even if previously controlled for age, FBT performance, or control questions. Adding first-person question may also significantly contribute to the model fit, but to a lesser extent than the second IT task.

That was exactly what we found. For the car-pen task, the three initial variables explained between 32% (Cox and Snell *R*^2^) and 43% (Nagelkerke *R*^2^) of variance with significant contribution of age included in the first step, and still important contribution of FBT included in the second step (see Table 2). Control and extensional questions were shown not to be significant together, which is not surprising, as children in all age groups performed very well and did not differ in this part of the tasks. Crucially, adding the police officer-dad task question to the model significantly improved the model fit, explaining an additional 14–18% of the variance, and the first-person question additionally explained about 4% of variance. However, when we look at the model parameters after the fourth or fifth step (see Table 3), adding these variables (especially the second IT) radically changed the model. Now, when controlling for these two variables, neither age nor FBT regression coefficients remain significant. This means that general developmental factors correlated with age and coarse knowledge of beliefs represented by FBT contributed to similar extent to both ITs, and thus their variances come into the common variance of both ITs. On the other hand, however, the common variance of both ITs is much more than that provided by age and FBT, and this additional part most probably represents some abilities or knowledge indispensable to solve IT.

**TABLE 2:** Predicting car-pen intensionality task IT1 (to third-person test question): hierarchical logistic regression.

Model	Variables in the model	*b*	Wald	Exp(*b*)	Significance of improvement χ^2^	Model summary		
						Cox and Snell *R*^2^	Nagelkerke *R*^2^	Model χ^2^
1	Age	0.099	27.811***	1.104	38.188***	0.273	0.363	38.188***
2	Age	0.078	15.581***	1.082	8.192**	0.321	0.427	46.380***
	FBT	1.543	7.639**	4.680				
3	Age	0.078	15.576***	1.082	0.061	0.321	0.428	46.441***
	FBT	1.547	7.669**	4.697				
	IT1 (C-E combined)	0.090	0.061	1.095				
4	Age	0.046	3.479	1.047	27.270***	0.459	0.612	73.710***
	FBT	−0.141	0.029	0.869				
	IT1 (C-E combined)	−0.342	0.578	0.710				
	IT2 (third-person test question)	3.270	19.392***	26.324				
5	Age	0.024	0.748	1.024	7.956**	0.494	0.658	81.666***
	FBT	−0.279	0.105	0.756				
	IT1 (C-E combined)	−0.285	0.367	0.752				
	IT2 (third-person test question)	2.995	14.901***	19.986				
	IT1 (first-person test question)	1.833	7.367**	6.252				

FBT, false belief task; IT1, car-pen task; IT2, police officer-dad task; C, control question, E, extensional questions. **p < 0.01, ***p < 0.001.

**TABLE 3:** Predicting police officer-dad intensionality test IT2 (to third-person test question): hierarchical logistic regression.

Model	Variables in the model	*b*	Wald	Exp(*b*)	Significance of improvement χ^2^	Model summary		
						Cox and Snell *R*^2^	Nagelkerke *R*^2^	Model χ^2^
1	Age	0.124	32.407***	1.132	51.003***	0.346	0.462	51.003***
2	Age	0.099	17.102***	1.104	24.231***	0.466	0.622	75.234***
	FBT	3.008	17.295***	20.252				
3	Age	0.100	17.178***	1.106	0.332	0.467	0.624	75.566***
	FBT	2.933	15.829***	18.786				
	IT1 (C-E combined)	–0.242	0.329	0.785				
4	Age	0.072	6.344	1.075	28.108***	0.579	0.772	103.674***
	FBT	3.270	11.161***	26.306				
	IT1 (C-E combined)	–0.594	1.434	0.552				
	IT2 (third-person test question)	3.323	20.184***	27.730				
5	Age	0.051	2.872	1.052	4.418*	0.594	0.793	108.092***
	FBT	2.929	7.811**	18.711				
	IT1 (C-E combined)	–0.722	1.936	0.486				
	IT2 (third-person test question)	3.286	17.262***	26.733				
	IT1 (first-person test question)	1.545	4.222*	4.687				

FBT, false belief task; IT1, car-pen task; IT2, police officer-dad task; C, control question; E, extensional questions. *p < 0.05, **p < 0.01, ***p < 0.001.

The model for the police officer-dad task gave similar results except that age and FBT contribution decreased, but remained at least close to significant even after adding to the model the results of the car-pen task (Table 3). Nonetheless, the result of the car-pen task remains the strongest predictor in this model, much above any other variable. Thus, we need to explain a significant part of the variance of IT performance with reference to the abilities that are common to two superficially different tasks, but which go far beyond either general-developmental factors or coarse-grained knowledge of what beliefs are. We will discuss these factors in greater detail in the general discussion, after presenting the results of Experiment 2.

**TABLE 4:** Distribution of correct answers to false belief and intensionality tests with two and four stickers.

	Age			
Trial	4	5	6	Total
FTB 2-sticker	18(72.0%)	20(80.0%)	23(92.0%)	61(81.3%)
FTB 4-sticker	15(60.0%)	17(68.0%)	20(80.0%)	52(69.3%)
IT 2-sticker	7(28.0%)_a_	10(40.0%)	22(88.0%)	39(52.0%)
IT 4-sticker	5(20.0%)_b_	9(36.0%)	21(84.0%)	35(46.7%)

*FBT, false belief task; IT, intensionality task. Each age group *n* = 25, total *N* = 75.*

### Discussion

In Experiment 1, we replicated typical results in both false belief and intensionality tests. As in the previous studies, only a few 3-year-olds, about half of the 4-year-olds, and most of the 5- and 6-year-olds passed the unexpected contents version of FBT. Moreover, also similarly to the previous studies, the performance in IT was delayed by about a year in comparison to FBT (cf. Apperly and Robinson, 1998, 2001, 2003; Hulme et al., 2003).

What is new in our results is that we have demonstrated that (1) the lag between IT and FBT depends neither on the form of the crucial information conveyed to the child (narrative vs. enactive, cf. the police officer-dad vs. car-pen task), nor on (2) the first- vs. third-person form of the test question. At the same time superficially different IT tasks require mostly the same cognitive conceptual abilities, which seem to be specific to understanding opacity.

Our results also show the high reliability and validity of the task designed to test intensionality. First, there was a high consistency of answers despite the clear difference in the form of information conveyed to the child. Second, it was not difficult to understand the task settings and to memorize crucial information: In all age groups, the ratio of correct answers to the control questions and extensional questions was high (at least 80% or more), and correlated neither with age nor performance in the test question.

Close comparison of the performance in the intensionality and false belief tests also showed that false belief understanding not only precedes understanding of opacity, but also that it is a prerequisite for it. Almost all children in the three younger groups (96%) who passed IT, also passed FBT. Passing FBT explains a significant part of the variance of both ITs, even when age is partialed out, as demonstrated in the logistic regression models. This leads us to the conclusion that FBT appeals to some conceptual capacities that are necessary but not sufficient to pass IT. Unfortunately, Experiment 1 does not reveal the nature of any additional cognitive competences needed to understand opacity. As many researchers have suggested, the main problem children have with intensionality tests may be that two names refer to the same object, and not in the opacity of meaning itself. Experiment 2 was designed to approach this problem more directly.

In Experiment 2, we modified both false belief and intensionality tasks in a way that neither test question required a nominal reference to the object. Moreover, we introduced the problem of multiplicity of reference not only into the intensionality task, but also into the false belief task. We preserved the scenarios of FBT (chocolates-pencils) and IT (car-pencil), but the children’s task was now to put some stickers representing the object’s identity on a sheet of paper, rather than to give their answers verbally. We designed two versions of the task: In one of them, the child’s task was to choose between two stickers while in the second version there were four different stickers, two for each possible choice. The child always had to select all correct stickers. In this way, we introduced multiplicity of label choices for the same object in both tasks (FBT and IT).

We considered three options. Firstly, if the problem with the opacity test lies in verbal reference (or, broader, in verbal demands), the two-sticker version should significantly improve children’s performance in IT, as only iconic reference is required in this case. Secondly, if it is the case that the main difficulty in intensionality tests lies in two names referring to the same object, but not in understanding opacity of meaning itself, then we could expect that the four sticker condition, which introduces dual label problem to the FBT should radically decrease children’s performance, bringing it closer to IT (to which dual label problem is inherent). Thirdly, if the problem is a conceptual one (and therefore it is present in IT, but not in FBT, regardless of the number of stickers), we can expect a constant (about one year) lag between FBT and IT performance in both two- and four-sticker.

## Experiment 2

### Method

#### Participants

Seventy-five children, divided into three age groups: 4-, 5- and 6-year-olds participated, aged 4;1–6;9. Participants formed three age groups of 25 children: 4-year-olds (mean age: 4;5, range: 4;1–4;11); 5-year-olds (mean age: 5;5, range: 5;1–5;10); and 6-year-olds (mean age: 6;5, range: 6;1–6;9). None of the children had participated in Experiment 1. Participants came from middle-income, urban families, from three different kindergartens in an average-size city in Poland. Written consent permissions were collected from the children’s parents before the study. We decided to skip the 3-year-olds as the performance on FBT and IT for this age group is usually low and does not differ significantly.

#### Tasks and Procedure

Four tasks were administered to each participant. The tasks were adapted from Experiment 1 (the unexpected contents version of FBT, and the car-pen IT). However, children were asked to put some stickers on a sheet of paper that illustrated their selected option, rather than to answer verbally. Each task had two versions: a two- and four-sticker one. While in the two-sticker version each option was represented by a single sticker, in the four-sticker versions each option was represented by two different stickers (colored and gray-scale ones). About half the participants completed the two-sticker version first; the remaining participants completed the four-sticker version first.

##### False belief tasks

**FIGURE 1:**
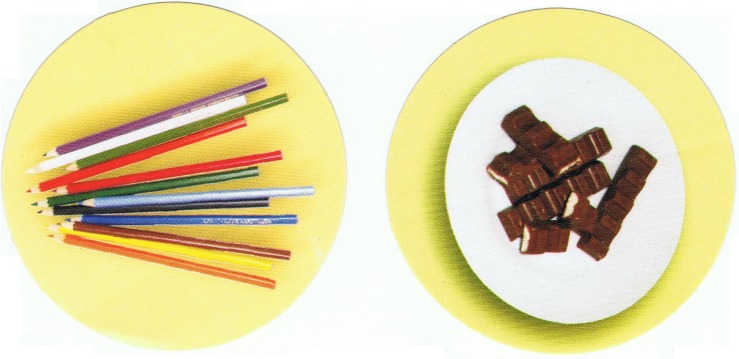
Stickers used in the two-sticker version of the false belief task.

**FIGURE 2:**
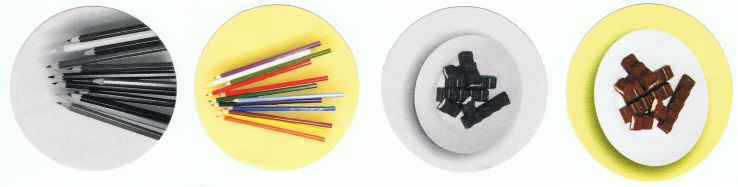
Stickers used in the four-sticker version of the false belief task.

The materials and procedure were modeled on the unexpected contents FBT used in Experiment 1. However, once the second experimenter left the room, two circle-shaped stickers were given to the child: one with a color picture of Kinder chocolates, and the other with a color picture of colored pencils (two-sticker version) (Figure 1). Alternatively, the child was given four stickers: two colored and two grayscale pictures of Kinder-chocolates and pencils (Figure 2)^4^. Then the child was asked: *“Which stickers will X choose to stick onto the box?”* (where X stands for the second experimenter’s name). Note that the word “stickers” was always in plural to prompt that multiple stickers might be used.

##### Intensionality tasks (car-pen task)

**FIGURE 3:**
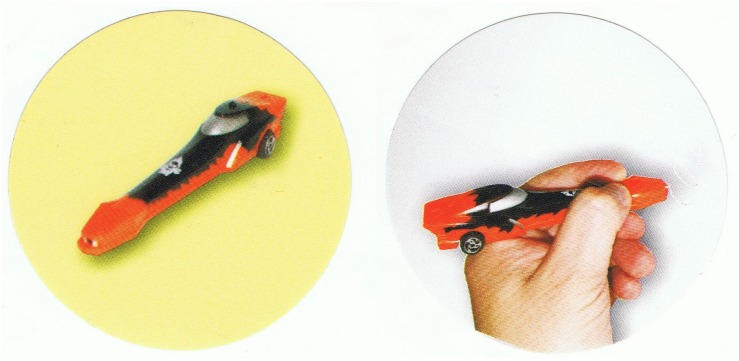
Stickers used in the two-sticker version of the intentionality test.

**FIGURE 4:**
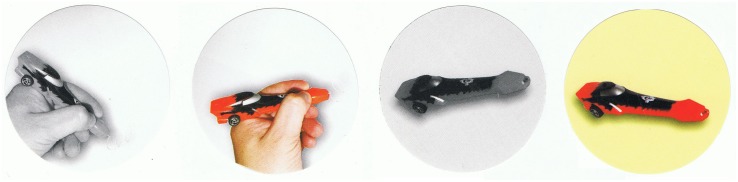
Stickers used in the four-sticker version of the intensionality test.

The task was adapted from Experiment 1, with the same modification as in FBT. Once the second experimenter left the room, the child was given two or four round stickers representing both object identities (car and pen), colored in the two-sticker version and colored and gray-scale in the four-sticker version (Figures 3,4). Then the child was asked which stickers would X stick onto the box where the car-pen was placed (the question wording was the same as in FBT). There were no control, extensional, and first-person questions asked in Experiment 2.

##### Scoring

In the two-sticker tasks, the answer was marked as correct if the child selected only one sticker corresponding to the primary identity of an object: pencils (FBT) or car (IT). In the four-sticker tasks, a correct answer was counted if the child selected both – colored and grayscale – stickers corresponding to the object’s primary identity. A more detailed analysis of the specific patterns of erroneous choices was also performed.

### Results

#### False Belief Tasks

The distribution of all four tests is summarized in Table 4. Because we did not include 3-year-olds in Experiment 2, no significant age differences in FBT performance were found this time [χ^2^(2) = 3.34, *p* = 0.189, *V* = 0.211, two sticker version; χ^2^(2) = 2.38, *p* = 0.304, *V* = 0.178, four-sticker version]. Children of all three age groups generally performed above chance in FBT with two stickers (all ps < 0.05, two-tailed binomial probability). The performance in the four-sticker version was slightly worse (however, in the entire sample and in 6-year-olds significantly above chance, two-tailed binomial *p* < 0.001, and *p* < 0.004, respectively; for collapsed 4- and 5-year-olds *p* = 0.065). However, the difference between the two- and four-sticker version does not approach significance in either age group, or in the entire sample (all ps > 0.65, McNemar χ^2^), and the convergence between these two measures is quite high (Yule’s φ = 0.72, *p* < 0.001). Importantly, neither the two-sticker version nor the four-sticker version diverge significantly from the results of standard unexpected contents task with verbal answer in Experiment 1 (two-tailed Fisher exact probability: *p* = 1.000, and *p* = 0.456, respectively).

#### Intensionality Tasks

Contrary to FBT, a significant age effect was found both for two- and four-sticker version of the IT [χ^2^(2) = 20.192, *p* < 0.001, *V* = 0.519, two sticker version; and χ^2^(2) = 22.20, *p* < 0.001, *V* = 0.545, four-sticker version]. Only 6-year-olds’ performance was above chance (exact binomial: *p* < 0.001, two-sticker version; and *p* < 0.001, four-sticker version). The results of the two versions were highly convergent (Yules φ = 0.63; *p* < 0.001), and in none of the groups did performance in the two- and four-sticker versions differ significantly (all ps > 0.850, McNemar χ^2^ test, two-tailed). Such performance level does not diverge significantly from the car-pen task results in Experiment 1 (two-tailed Fisher exact probabilities, respectively, for two- and four-sticker versions: *p* = 0.572 and *p* = 0.237 for 4-year-olds; *p* = 0.108 and *p* = 0.060 for 5-year-olds; *p* = 0.990 and *p* = 0.990 for 6-year-olds) (see Table 4).

#### Comparison Between False Belief Task and Intensionality Task With Stickers

Our critical hypotheses concerned the differences between FBT and IT. If the younger children’s problem with opacity lies in the multiplicity of reference, we may expect that the four-sticker task version should significantly worsen performance in FBT as it introduces multiple reference. It should not, at the same time, affect the performance in IT, to which the multiple reference problem is inherent. On the other hand, if the problem lies in the verbal form of the task, using stickers instead of verbal reaction should improve IT performance. However, if the problem with opacity is conceptual in nature, the difference between FBT and IT should remain stable independently of the task version. Although we have observed some weak tendency toward decreasing performance in the four-sticker FBT, it was not significant in any age group. The difference between FBT and IT remained stable and significant in both two- and four-sticker tasks in the two younger groups, and disappeared in 6-year-olds in the same manner as in the standard tasks in Experiment 1 (two-tailed McNemars χ^2^: all ps < 0.05 for 4- and 5-year-olds; *p* = 0.990, two-sticker version; and *p* = 0.880, four-sticker version for 6-year-olds). Moreover, using stickers instead of verbal labels did not improve performance in either test even in the two-sticker version (see Table 4).

#### Analysis of Specific Patterns of Choices in the Four-Sticker Tasks

There are apparent differences in the wrong choice patterns between FBT and IT. While more than a half (12 out of 23) of the wrong choices in FBT consistently referred to a single identity, the same was the case for only six out of 40 in IT. The difference is highly significant [McNemar χ^2^(1) = 10.76, *p* < 0.001). The modal pattern of wrong choices in FBT was selecting both stickers referring to reality (11 out of 23, none of the other patterns exceeded four out of 23]. In IT, the modal (22 out of 40 erroneous choices) was selecting all four stickers (only one single choice of this pattern was found in FBT). This distribution of error patterns clearly shows that processing opacity is guided by partially different conceptual structures than processing false beliefs.

#### Hierarchical Logistic Regression Model

Hierarchical logistic regression was used to determine to what extent the four-sticker IT as the dependent variable can be explained by age, with the four-sticker FBT and the two-sticker IT as predictor variables. With this we hoped to see whether the number of stickers was a good predictor in both IT and FBT. As indicated in Table 5, adding answers from the two-sticker IT test to the third model explains at least 10% of the variance, beyond the one explained by age and FBT (10.0% Cox and Snell *R*^2^, 13.3% Nagelkerke’s *R*^2^), which is more than the FBT-accounted variance. Testing the logistic regression parameters of variables in the final model demonstrated that it was age (*b* = 1.085, *p* < 0.001) and the two-sticker IT (*b* = 2.259, *p* < 0.001) that were statistically significant. After introducing the two-sticker IT into the model, the four-sticker FBT ceased to be significant as a result of a high variance explanation that was shared with the two-sticker IT (*b* = 0.752, *p* = 0.313).

**TABLE 5:** Predicting to IT [4 sticker task (test question)]: hierarchical logistic regression.

Model	Variables in the model	*b*	Wald	Exp(*b*)	Significance of improvement χ^2^	Model summary		
						Cox and Snell *R*^2^	Nagelkerke *R*^2^	Model χ^2^
1	Age	1.527	16.999***	4.605	22.301***	0.257	0.343	22.301***
2	Age	1.520	15.359***	4.572	6.274*	0.317	0.423	28.575***
	FBT 4-sticker	1.573	5.621*	4.821				
3	Age	1.085	6.478*	2.958	11.844***	0.417	0.556	40.419***
	FBT 4-sticker	0.752	1.018	2.120				
	IT 2-stickers	2.259	10.846***	9.569				

FBT, false belief task; IT, intensionality task. *p < 0.05, ***p < 0.001.

### Discussion

In Experiment 2, we modified both FBT and IT in a way that neither the test question nor the answers required nominal reference to the object. The problem of multiplicity of reference was also introduced to both tasks. We preserved the scenarios of both FBT (chocolates-pencils) and IT (car-pen) from Experiment 1, but instead of verbally indicating the correct answer, the child’s task was to put on a sheet of paper some stickers representing object identity. The two versions of the tasks–two-sticker and four-sticker–were administered to all participants. The assumption was that this manipulation induced multiple-name reference to the same object (reference resolution), which is normally a feature distinguishing IT from FBT. This is important because there has not been any study so far that aimed to directly show whether the difference in children’s performance on the standard FBT and IT has its source in reference resolution, or rather in conceptual distinction required specifically to understand opacity of representation. Our results show that the difference between FBT and IT remains stable and significant in both two- and four-sticker tasks in the two younger groups and disappears in six-year-olds in the same manner as in the standard tasks from Experiment 1.

There were clear and significant differences in the patterns of incorrect answers between FBT and IT. While more than half of the incorrect answers in FBT consistently referred to the single identity, only 15% did so in IT. The modal pattern of incorrect answers in FBT was selecting both stickers referring to reality, while in IT the modal was to select all four stickers (only one single choice of this pattern was found in FBT). This distribution of error patterns, together with the hierarchical logistic regression model explaining performance in the four-sticker version of IT, clearly shows that processing opacity and false beliefs are guided by partly different conceptual structures.

The introduction of multiple terms or labels referring to the same object did not significantly decrease the level of performance in either test. The necessity of choosing two stickers (out of four) as correct ones only reduced a little the success rate in distinguishing between the two alternative modes of presentation of a given object. Further, children who succeeded in the two-sticker version did well with four stickers as well, regardless of the kind of test. Even more importantly, we have shown that the number of stickers does not significantly influence the ratio of FBT to IT.

In light of the above, an explanation other than the one that exclusively relies on linguistic or executive complexity of dual reference is needed for the IT-FBT performance gap. In our opinion, the only known possibility that has not been ruled out by our results is that children have a conceptual problem with separating two modes of object presentation and associating one particular mode of presentation with a particular person. Children reject substitution of description more readily when they know that the protagonist’s conceptualization (belief) is false than when both conceptualizations are true; it is more difficult for them to reject a substitution of description when they know the protagonist’s conceptualization (belief) is true under an alternative description. The introduction of multiple labels for one choice does not in itself modify these differences.

## General Discussion and Conclusion

One of the general preconditions of passing FBT is the understanding that beliefs might not coincide with reality. More specifically, passing FBT requires recognizing that a belief can be wrong, i.e., that “beliefs, by their nature, are subject to correction, and that to correct a false belief requires representing that the belief is false” (Laurence and Margolis, 2012, p. 313). It follows then that the notion of belief which the child possesses at the time of passing FBT incorporates the content of epistemic evaluation (i.e., ascription of truth value to the belief), representing and misrepresenting (notion that beliefs are always about something, but may erroneously represent its object) and fitting direction (which in the case of belief is from the mind to the world).

Earlier studies, as well as our present one, show that children find it harder to pass the opacity test than the false belief test. Having weakened the possibility that this difficulty lies in executive or linguistic demands (e.g., double reference resolution), we believe we need to shift our attention to the concept of belief itself (i.e., learning it) and ask why this concept allows young children to pass FBT, but not IT, at a certain age. To answer that question, we should look at the two tasks from the perspective of the conceptual conditions that need to be satisfied if the child is to pass them. In other words, what needs to be investigated, we believe, are the additional elements of the concept of “belief” that need to be acquired by the child so that she can transition from understanding beliefs can be false to understanding the opacity that characterizes propositional attitudes. This transition will allow them cope with those situations where – as Lalonde and Chandler, 2002 point out – people hold two different opinions about the same object.

(i) To pass IT, the child has to be conceptually able to distinguish between knowing two true descriptions of one and the same object. To make that distinction, the child cannot rely on the differentiation between representing and misrepresenting, or even less so on the one between representing and non-representing. Applying this dichotomy is sufficient for FBT simply because when solving FBT, we deal with two conflicting alternative descriptions. In order to pass IT, however, it is not enough to track whether the belief represents or misrepresents the object (referent), or even how the person evaluates the belief (whether they understand a false belief as a true one).

(ii) Conversely, in IT, the content of the propositional attitude has to be specified in terms of a point of view, aspectual shape, or perspective, but not only based on the epistemic evaluation of something as true or false. Only the category of perspective does allow the child to distinguish two different, but equally legitimate terms for the same object. It is crucial, however, to differentiate *perspective taking* or the *point of view* in the current sense from the *perceptual perspective*. Understanding perceptual perspective does not require understanding that parallel true representations of the same object may exist independently. Only one perceptual perspective may be true for one person at one time. Understanding that perceptual representation depends on the perceptual access, which in turn may depend on the point of view, is related to *representing* and *misrepresenting* distinction, and thus emerges together with understanding false beliefs.

(iii) The next difference is best demonstrated by the problem of closure for the epistemic functor “*X* knows that…”. Let us assume that (1) *X* knows that *p* and that (2) *q* belongs to Cn(p), where Cn means a logical consequence operation. And finally, let us ask if (3) *X* knows that *q*. Clearly, not necessarily: (1) and (2) may be true, and (3) may be false because if person *X* knows that *p*, she does not need to know all the logical consequences of this statement. In logic, this issue is resolved by positing the logical omniscience of epistemic subjects. This is, however, a psychologically unrealistic solution. It seems that when children have to judge whether *X* knows that *q*, they close the epistemic functor – if *p* is true and *q* is a logical consequence of *p*, they assume that since *X* knows that *p*, she also has to know that *q*. In FBT, knowing that the belief “there are chocolates in the box” is false, the child reaches the following conclusion: “if the person has a false belief in mind, then she does not know that there are pencils in the box.” The key issue seems to be that the knowledge applied to block the closure of the epistemic functor is *de re* knowledge, (“there are chocolates in the box” is false). In IT, both sentences taken extensionally (“there is a car in the box” and “there is a pencil in the box”) are true. Thus, relying on extensional knowledge, we do not differentiate one belief from the other.

Considering all the above differences between the false-belief and intensionality problems, and the kind of mistakes that children who pass FBT make on IT (choosing two stickers instead of one), it seems that they conceive of mental content ascribed to another person in terms of truth and reference conditions. They seem to prioritize the identity of reference over the identity of mental content (point of view or perspective). Hence, if the two propositions “The police officer helped Anna” and “Anna’s dad helped Anna” share the same truth value and each of them has the same reference (the police officer is Anna’s dad in this case), children think that even in intensional belief contexts, it is still possible to substitute co-referential terms. Applying this rule, which leads to wrong substitutions in IT, greatly limits understanding that beliefs are always held under particular descriptions, not others (Searle, 1992, p. 131).

However, we do not espouse the claim that there are two models – a word-referent one and a word-sense-referent one – that children passing FBT and IT apply, respectively. It is possible from our perspective that at the time of coping with FBT, children already use a representational theory of mind (Kamawar and Olson, 1999, 2011). Rather, we suggest that both FBT and IT involve a word-intension-referent model, but in FBT the “intension” is always qualified as either “true” or “false.” In IT, on the other hand, on top of the true-or-false parameter, there is also knowledge needed that one can know something only under one of many true presentations. Therefore, the key point is whether sense is seen as one-dimensional (FBT: only true vs. false; what might be viewed as a more fundamental epistemic psychology), or two-dimensional (IT: true yet different; what might be understood as an advanced epistemic psychology). However, we still think that we have a solid reason to propose that this difference between FBT and IT is not about reference resolution because both involve multiple perspectives. Perspective taking requires the additional competence of distinguishing the intensions under which the object is known (e.g., dad or police officer), which is more than just knowing if the person registered the object or not. Registration of an object itself does not tell us *how* the person grasps the object (e.g., as “dad” or “police officer”).

Overcoming the epistemic limitation requires the child to think in a subtler way, using a concept of belief that allows for considering the intension under which a given belief is presented, not only its truth value. Once the child has acquired such an extended concept of belief, he/she is able “to exploit fine-grained representations, representations exhibiting semantic opacity” (Heil, 1992, p. 203), which can be considered a step toward a more advanced or interpretive theory of mind.

Only thanks to this can the child go beyond thinking about the referent directly, and think of it as represented in a particular way. This development may be interpreted as a gradual transition from coarse-grained content to fine-grained content or from unsophisticated knowledge to sophisticated one (McGinn, 1989). This entails abandoning the standard way of viewing what concepts are. It is important to bear in mind that in both cases of belief concept we still deal with the same kind of competence based on understanding what a belief is and how it is constrained by the knowledge held by the protagonist. And finally, in both cases, it is necessary to distinguish between the extensional object (a police officer helping Anna to cross the street, or actual object’s location in FBT), and the intensional context in which the extensional content is expressed (the protagonist’s belief about the person who is helping Anna to cross the street, or about the object location in FBT).

Considering these similarities, we refrain from espousing the interpretation that the process of transition between the ability to pass FBT and the ability to pass IT is a transition between two radically different systems of theory of mind. However, we are still inclined to recognize that false belief problems and opacity problems are partly distinct classes of belief reasoning (cf. Apperly and Robinson, 2003). We have dismissed as unreliable the hypothesis that intensionality tests are more difficult only due to extraneous performance factors (cf. Rakoczy et al., 2015; Rakoczy, 2017). We claim that the difference between the two tasks is a real and involves a conceptual difference.

We assume that after reaching the stage of the basic concept of belief as subjective representational state around the child’s fourth birthday, ToM still unfolds in a piecemeal fashion and does not constitute a unitary phenomenon (Lalonde and Chandler, 2002; Apperly, 2011; Wellman, 2014). From that moment on, the development takes the form of a transition from coarse-grained content to fine-grained content. The changes become continuous in their nature, and are about complementing the present state of knowledge with additional elements, which allow the child to not only see new consequences (e.g., emotional consequences of beliefs), but also represent mental states in different ways (Lalonde and Chandler, 2002; Kamawar and Olson, 2009, 2011). In other words, we may characterize this process as an “ongoing enrichment” that consist in expanding the content of particular concepts, which enables the child to add new, more sophisticated principles of mental states attribution to the already existing abilities, and proceed to develop even more complex socio-cognitive skills in their life after age 5 (Wimmer and Hard, 1991; Carpendale and Chandler, 1996; Lalonde and Chandler, 2002; Lagattuta et al., 2010). They are not, however, radically new concepts. The specificity of the concept of belief consists in the fact that its components of falsity and opacity are not equivalent and cannot be interchanged freely in all belief attributions. The enrichment of the notion of belief occurs when the child learns the fine-grained principles of individuation of beliefs; namely, that beliefs always incorporate an “aspectual dimension.” Children realize that beliefs are not only about something, but that they involve a certain point of view, i.e., a specific way of rendering an object, and consequently children start to comprehend that having access to an object under one description does not ensure access to that object under all descriptions.

## Data Availability Statement

The datasets generated for this study are available on request to the corresponding author.

## Ethics Statement

The studies involving human participants were reviewed and approved by the Komisjȩ Etyki Badań Naukowych, Katolicki Uniwersytet Lubelski Jana Pawła II Al. Racławickie 14, 20–950 Lublin. Written informed consent to participate in this study was provided by the participants’ legal guardian/next of kin.

## Author Contributions

AG was responsible for the research idea, research design, data collection, interpretation of data analysis, and writing up the results. MH worked on the research idea, interpretation of data analysis, and writing up the results. OG analyzed the statistical data. MC collected the data and interpreted the data analysis.

## Conflict of Interest

The authors declare that the research was conducted in the absence of any commercial or financial relationships that could be construed as a potential conflict of interest.

## Footnotes

1 We understand o*pacity* and *intensionality* synonymously in the paper and use them interchangeably. Strictly, opacity or intensionality means the content of a mental representation (e.g., beliefs), the particular mode of presentation or Fregean sense through which the representation achieves its reference. Thus, to understand opacity or intensionality of other minds is to understand that they represent reality always through some particular presentation, e.g., historically Venus was represented as the morning star and the evening star, and only later was it discovered that the two actually refer to the same celestial body.

2 The same criticism applies to the procedure of the intensionality test used in the newest study by Oktay-Gür et al. (2018), although some additional issues may be raised in this case.

3 We also explored whether there were any differences between answers to these questions: “Does X who has left the room know/think that there are pencils in the box?” and “What does X who left the room think is in the box, pencil or chocolate?”. Hence, we did not merely ask whether X knows or not, but rather whether X knows what is inside and then suggesting the answer. Moreover, the sticker version of our test does not ask think or know questions, but rather “Which stickers will X (who has left the room) choose to stick onto the box?”. Importantly, the response patterns to the sticker question and the verbal questions are virtually identical. We therefore conclude that in both of these versions (verbal and sticker), the child needs to refer to the other person’s belief state thus both of the questions are appropriate for FBT.

4 Devising the sticker version that would limit the verbal load (esp. understanding synonyms), we searched for such a change in how the stickers looked which would mirror the chocolate-something sweet difference in the verbal tasks. Our rationale in changing the colors was based on the extant developmental knowledge that difference in color is an important one for children. Additionally, other studies of ToM also used color change (of a mug) for measuring the ability to attribute mental states in 3- and 4-year-olds (Flavell et al., 1990). We reasoned, therefore, that for a preschooler, changing the color from red to gray is similar to changing “chocolate” to “something sweet“. In the future, it would be of course illuminating to conduct a study using not only stickers of different colors but also stickers with different kinds of objects (for example, different shape of the object depicted, or different brand), but we believe that there are strong motivations for our current methodology regardless.
